# Clinical and pathologic characteristics of inflamed juvenile conjunctival nevus and its treatment with immunosuppressant eye drops

**DOI:** 10.1007/s10384-024-01140-9

**Published:** 2024-11-16

**Authors:** Ayako Tomoda, Kaoru Araki-Sasaki, Hiroto Obata, Shinji Ideta, Masahiko Kuroda, Koji Fujita, Yasuhiro Osakabe, Kanji Takahashi

**Affiliations:** 1 Department of Ophthalmology, Kansai Medical University, 2-5-1 Shinmachi, Hirakata, 573–1191 Osaka Japan; 2 Department of Ophthalmology, Saitama Medical University, Saitama, Japan; 3 Ideta Eye Hospital, Kumamoto, Japan; 4 Department of Molecular Pathology, Tokyo Medical University, Tokyo, Japan

**Keywords:** Allergic conjunctivitis, Anterior segment optical coherence tomography angiography, Conjunctival nevus, Conjunctival tumor, Tacrolimus

## Abstract

**Purpose:**

To clarify the clinical and pathologic findings of 7 patients with inflamed juvenile conjunctival nevus (IJCN) treated with tacrolimus.

**Study design:**

Retrospective study.

**Subjects and Methods:**

The medical records of 7 male patients diagnosed with IJCN between February 2007 and October 2022 at the Kansai Medical University Hospital and Ideta Eye Hospital were retrospectively reviewed. The patients’ mean age was 11.3 (8–20) years, and the average follow-up period was 18 months. The patients underwent anterior segment optical coherence tomography (AS-OCT) and anterior segment optical coherence tomography angiography (AS-OCTA). All the patients were treated with steroids or immunosuppressant eye drops, or both. Histopathologic examinations were performed on specimens resected from 2 patients.

**Results:**

The clinical characteristics of IJCN were as follows: (1) presence of cysts within a pigmented conjunctival mass, (2) tumor accompanied by feeder vessels, and (3) predominantly found in boys with allergic diseases. AS-OCT and AS-OCTA revealed the presence of a cystic structure with poor vascular signals within it and numerous vessels distributed on the sclera underlying the lesion. Immunosuppressant eye drops relieved the congestion, diminished the inflow vessels, and induced shrinkage of the cysts in all the patients. Histopathologic examinations revealed infiltration of inflammatory cells such as lymphocytes and macrophages and low proliferative activity of melanocytes with no division pattern.

**Conclusions:**

This study clarified the clinical and histologic characteristics of IJCN. Noninvasive AS-OCT and AS-OCTA might be useful for diagnosing IJCN. The histopathologic findings of this study indicate that immunosuppressive and antiallergic agents can effectively bring about disease remission.

## Introduction

Conjunctival nevi are benign elevated lesions originating from melanocytes. Inflamed juvenile conjunctival nevi (IJCNs) are conjunctival nevi with inflammatory findings that are prevalent in the corneal limbus during childhood and adolescence [1]. IJCN develops on the bulbar conjunctiva, often with pigmentation, and enlarges rapidly with vascular invasion [2]. IJCN is a benign tumor; however, it must be differentiated from malignant melanoma in some cases because of its rapid enlargement [2–4].

Cysts are an important clinical finding that are used to diagnose conjunctival nevi [2, 5]; however, detecting cysts through slit-lamp examination in cases with hyperpigmentation and severe inflammation is difficult. Vascular invasion is a hallmark of IJCN that is also observed in patients with malignant melanoma. In these circumstances, surgical resection is sometimes performed for differential diagnosis [5, 6]. However, biopsy and surgical treatment are discouraged in children, and inadequate resection can cause recurrence [7]. Thus, characterizing the clinical and histopathologic findings for differential diagnosis using new instruments, such as anterior segment optical coherence tomography (AS-OCT) and anterior segment optical coherence tomography angiography (AS-OCTA), is necessary.

Histopathologically, IJCN involves infiltration of lymphocytes, eosinophils, and plasma cells into the nevus tissue, suggesting an association with allergic diseases. Exacerbation of allergic conjunctivitis results in rapid enlargement of the cysts over a short period of time [2, 6–10]. Thus, IJCN may be associated with allergic reaction, and the efficacy of steroid and antiallergic eye drops has recently been reported [11]. However, steroid therapy is not recommended in children aged younger than 9 years as it may induce an increase in intraocular pressure [12].

The aim of this study was to characterize new clinical findings of IJCN shown on AS-OCT and AS-OCTA by reviewing the clinical course of 7 patients. A further aim was to determine the efficacy of immunosuppressant eye drops as treatment for this disease.

## Subjects and Methods

This study included 7 eyes of 7 male patients with IJCN treated at the Kansai Medical University Hospital and Ideta Eye Hospital between February 2007 and October 2022. The patients’ average age was 11.3 years (8–20 years), and the average follow-up period was 18 months. All the patients underwent slit lamp microscopic examination. In addition, AS-OCT (SS-1000 CASIA; TOMEY) and AS-OCTA (PLEX Elite model 9000; Carl Zeiss) with a + 10D anterior lens was conducted in 2 patients. All the patients were given antiallergic, steroid, and immunosuppressant (0.1% tacrolimus and 0.1% cyclosporine) eye drops. Two of the 7 patients underwent excision of the lesion for cosmetic reasons after treatment with immunosuppressive drugs. The pigmented lesions were resected, and the inflow vessels were cauterized to prevent recurrence. Administration of antibacterial and steroid eye drops was commenced postoperatively. Histopathologic and immunohistochemical examinations were performed using the following antibodies: Melan A (mouse, clone A103, 1:50 dilution; Dako), Melanosome (mouse, clone HMB45, 1:100 dilution; Dako), Ki-67 (mouse, clone MIB-1, 1:400 dilution; Dako), S100 (polyclonal rabbit, 1:1000 dilution; Dako), CD3 antibody (polyclonal rabbit, ready-to-use; Dako), CD20 antibody (mouse, clone h-CD, 1:100 dilution; Dako), CD68 antibody (mouse, clone PG-M1, 1:200 dilution; Dako), and CD44 (mouse, clone DF1485, 1:50 dilution; Dako). Antigen retrieval for each antibody was performed using deparaffinized and hydrated slices; the slices were then incubated in blocking milk buffer followed by affinity-purified primary antibodies at room temperature. After incubation with a secondary alkaline phosphatase-conjugated antibody diluted in TBS (Tris-buffered saline), Fast Red substrate solution was applied for color development. Nuclear counterstaining was performed using Mayer’s hematoxylin solution, and the samples were observed under a microscope.

This study was conducted in accordance with the Declaration of Helsinki. Written consent was obtained from the patients undergoing treatment, and patients who had completed the treatment were provided with the opportunity to opt out. This study was approved by the ethics committees of the 2 institutions (approval numbers: 2022139 [Kansai Medical University] and IEH-2022001 [Idea Eye Hospital]).

### Representative case

**Fig. 1:**
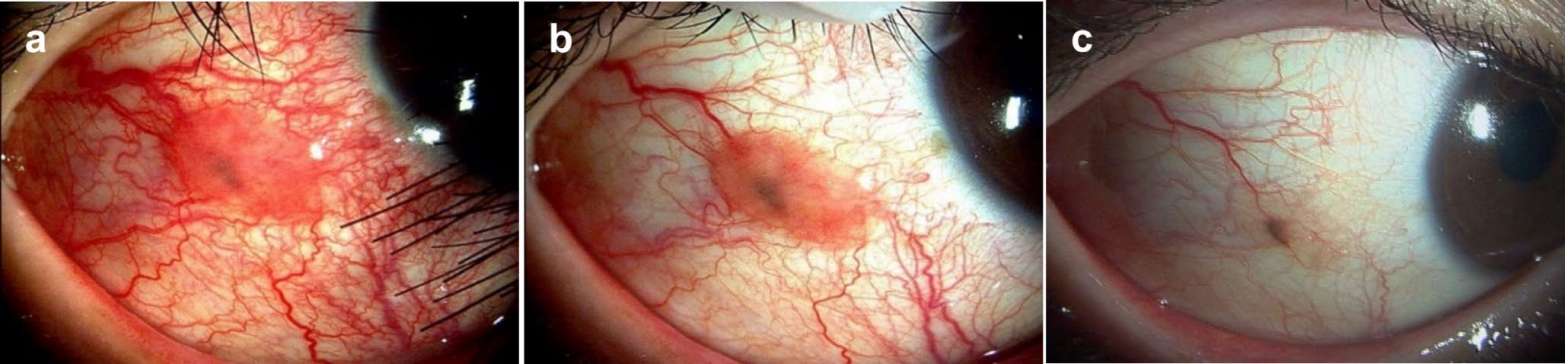
Slit-lamp photographs of the affected eye in case 1.**a:** Image acquired at the time of the initial visit to the clinic. A red elevated lesion (7 × 4-mm in size) with vessels can be observed on the right bulbar conjunctiva. Cysts are visible within the pigmented lesions. **b:** Image acquired after 4 months of treatment with olopatadine eye drops. The conjunctival hyperemia is resolved; however, the pathologic lesion persists. **c:** Image acquired after additional treatment with tacrolimus eye drops for 18 months. The inflammation and raised lesion are resolved

A 20-year-old man with a history of allergic conjunctivitis presented with pigmentation in the right bulbar conjunctiva that had increased in size over the years. He was referred to our hospital for a conjunctival tumor (Fig. 1a). Examination of the right bulbar conjunctiva revealed a 7 × 4-mm brown mass with feeder vessels and marked hyperemia. A cyst could be observed within the elevated lesion through the brown pigmentation. The patient was diagnosed with IJCN and given olopatadine eye drops 4 times a day for 4 months. A slight resolution of the hyperemia around the nevus was observed; however, the pathologic nodular lesion persisted (Fig. 1b). Administration of 0.1% tacrolimus eye drops twice daily was then started. Dexamethasone eye drops were also administered temporarily during the observation period owing to the incidence of vernal keratoconjunctivitis. Eighteen months after initiation of tacrolimus eye drop administration, the pathogenic region was resolved and the nutrient vessels were narrowed (Fig. 1c). The administration of tacrolimus eye drops was tapered off without exacerbation of the disease.

## Results

**Fig. 2:**
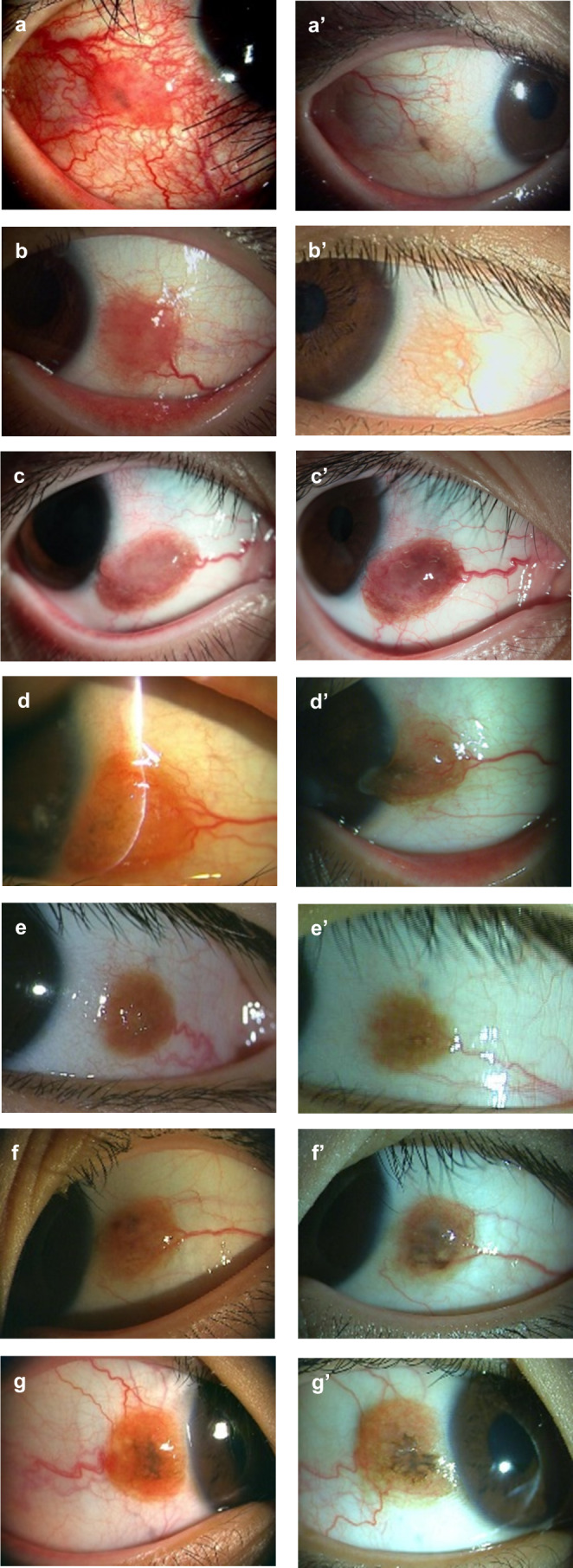
Slit-lamp photographs of all the cases at the initial examination. Images **a** to **g** correspond with cases 1 to 7 in Table 1. Elevated inflammatory lesions with hyperemia are visible in the conjunctiva near the limbus in all the patients, with remarkable vascular invasion. Internal pigmentation and cysts are present in all the cases. The lesions occur on the auricular side in 5 cases and on the nasal side in 2 cases. Images **a’**–**g’** are photographs of the anterior segment captured after each treatment period. Although the effect of treatment varies among the cases, slit-lamp examination reveals that the hyperemia has improved and the tumor diameter is reduced in all cases

Table 1 summarizes the anterior segment findings, background characteristics, clinical findings, follow-up period, and prognosis of the 7 patients. Figure 2 shows anterior segment photographs of the 7 cases at the time of the initial examination (Fig. 2a–g) and after the treatment (Fig. 2a’–g’). All the patients had had allergic conjunctivitis since childhood. Through the medical interviews, we discovered that more than half of the patients had a history of allergic rhinitis or atopic dermatitis. Therefore, we concluded that all 7 patients had a history of some form of allergic disease. Slit-lamp examination revealed that the pathologic regions were located near the corneal limbus (in 3 and 4 patients in the right and left eyes, respectively) and were more common in the bulbar conjunctiva (in 5 and 2 patients in the temporal and nasal conjunctiva, respectively). The presence of cysts within the tumors, large feeder vessels, and hyperpigmentation were frequently observed. Antiallergic, steroid, and immunosuppressant (0.1% tacrolimus or 0.1% cyclosporine) eye drops were administered to 1, 5, and 7 eyes (overlapped), respectively. The administration of immunosuppressant eye drops was continued for 10.1 months on average. Resolution of the hyperemia and reduction of the tumor diameter, determined on slit-lamp examination, was considered to indicate “improvement.” Worsening of the hyperemia and enlargement of the tumor diameter, determined on slit-lamp examination, was considered to show “recurrence.” The patients of cases 3 and 7 underwent surgical excision of the pathologic lesions after treatment with immunosuppressive agents for cosmetic reasons.

**Table 1:** Clinical characteristics of the 7 cases of inflamed juvenile conjunctival nevus

Case	Age, y/Sex	General allegic predisposition	Eye/Location	Cysts	Medical treatment	Duration of immunosuppressant eye drop prescription, mo	Prognosis for treatment	Elevation of IOP by steroid eye drop
1	20/M	Yes	OD/Temporal	Yes	0.1% Olopatadine 0.1% Dexamethasone 0.1% Tacrolimus	31	Improved^b^	No
2	11/M	Yes	OS/Temporal	Yes	0.1% Dexamethasone 0.1% Fluorometholone 0.1% Tacrolimus	16^a^	Improved but recurred^c^	No
3	14/M	Yes	OD/Nasal	Yes	0.1% Dexamethasone 0.1% Tacrolimus	4	Improved and resected	No
4	9/M	Yes	OS/Temporal	Yes	0.02% Fluorometholone 0.1% Cyclosporin	11	Improved	Yes
5	8/M	No	OS/Temporal	Yes	0.02% Fluorometholone 0.1% Cyclosporin	8	Improved	No
6	8/M	No	OS/Temporal	Yes	0.1% Cyclosporin	2^a^	Improved	No
7	9/M	Yes	OD/Temporal	Yes	0.1% Fluorometholone 0.1% Cyclosporin	3.5	Improved and resected	Yes

*IOP* intraocular pressure, *OD* oculus dexter, *OS* oculus sinister

^a^Administration of eye drops was interrupted based on self-judgment

^b^Improvement was considered to have occurred when, on slit-lamp examination, the hyperemia was resolved and the tumor diameter was smaller

^c^Recurrence was considered to have occurred when, on slit-lamp examination, worsening of the hyperemia and enlargement of the tumor diameter were determined

### AS-OCT and AS-OCTA imaging

**Fig. 3:**
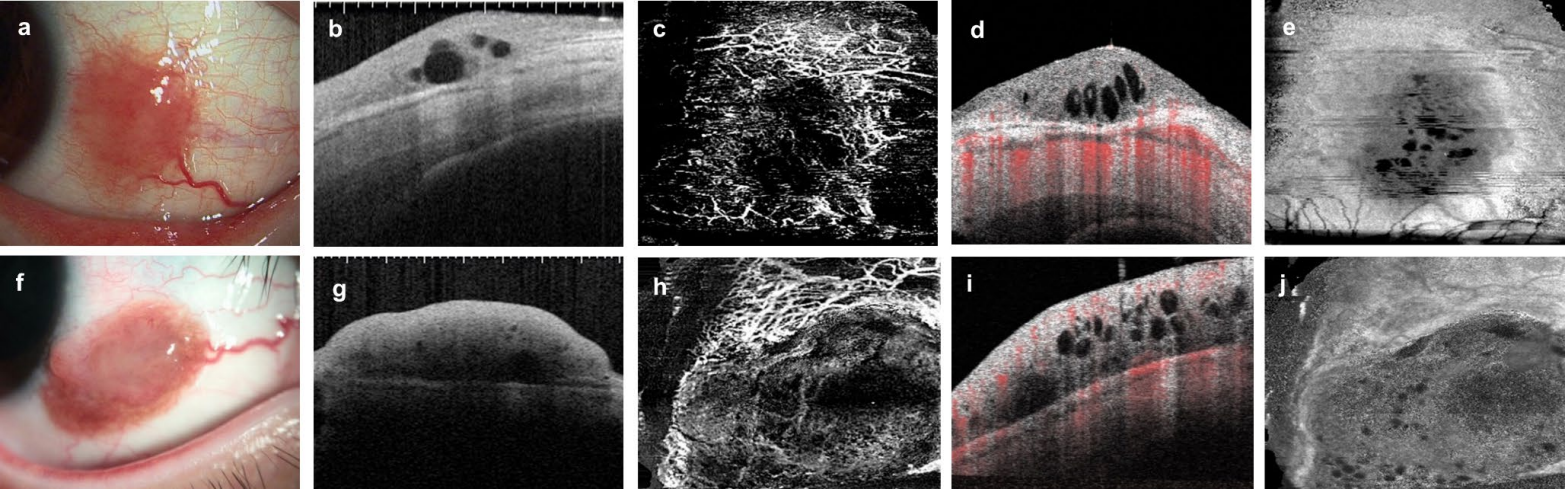
Anterior segment optical coherence tomography (**b**,** g)** and anterior segment optical coherence tomography angiography (**c–e**,** h–j)** images of cases 2 and 3. Images **a** to **e** show the outcomes of case 2, and **f** to **j**, the outcomes of case 3. **a**, **f:** Red raised lesions and large blood vessels are observed at the time of resection. **b**, **g:** Anterior segment optical coherence tomography (AS-OCT) scans show cysts within the mass and a clear boundary between the lesion and the sclera. **c**, **h:** In both cases, AS-OCT angiography reveals poor blood flow within the lesion. **d**, **i:** Of note is that the central B-scans of AS-OCT angiography show poor vascular invasion in the mass but abundant blood flow in the underlying sclera. **e**, **j:** Structural AS-OCT angiography images reveal multiple cysts within the lesion

Figure 3 presents the anterior segment photographs and AS-OCT and AS-OCTA scans of cases 2 and 3 (as listed in Table 1). AS-OCT scans (Fig. 3b, g) visualized multiple low-intensity luminal structures indicative of cysts within the mass. The basal portion of the lesion was clearly demarcated from the underlying sclera. Optical shadowing was observed posterior to the nevus in areas with significant pigmentation. However, the AS-OCTA scans (Fig. 3c, h) revealed poor blood flow within the lesion. The central B-scan AS-OCTA images (Fig. 3d, i) showed poor vascular invasion within the mass but also revealed abundant blood flow, primarily in the underlying sclera. The structural AS-OCTA images (Fig. 3e, j) revealed the presence of cysts.

### Immunohistochemistry

**Fig. 4:**
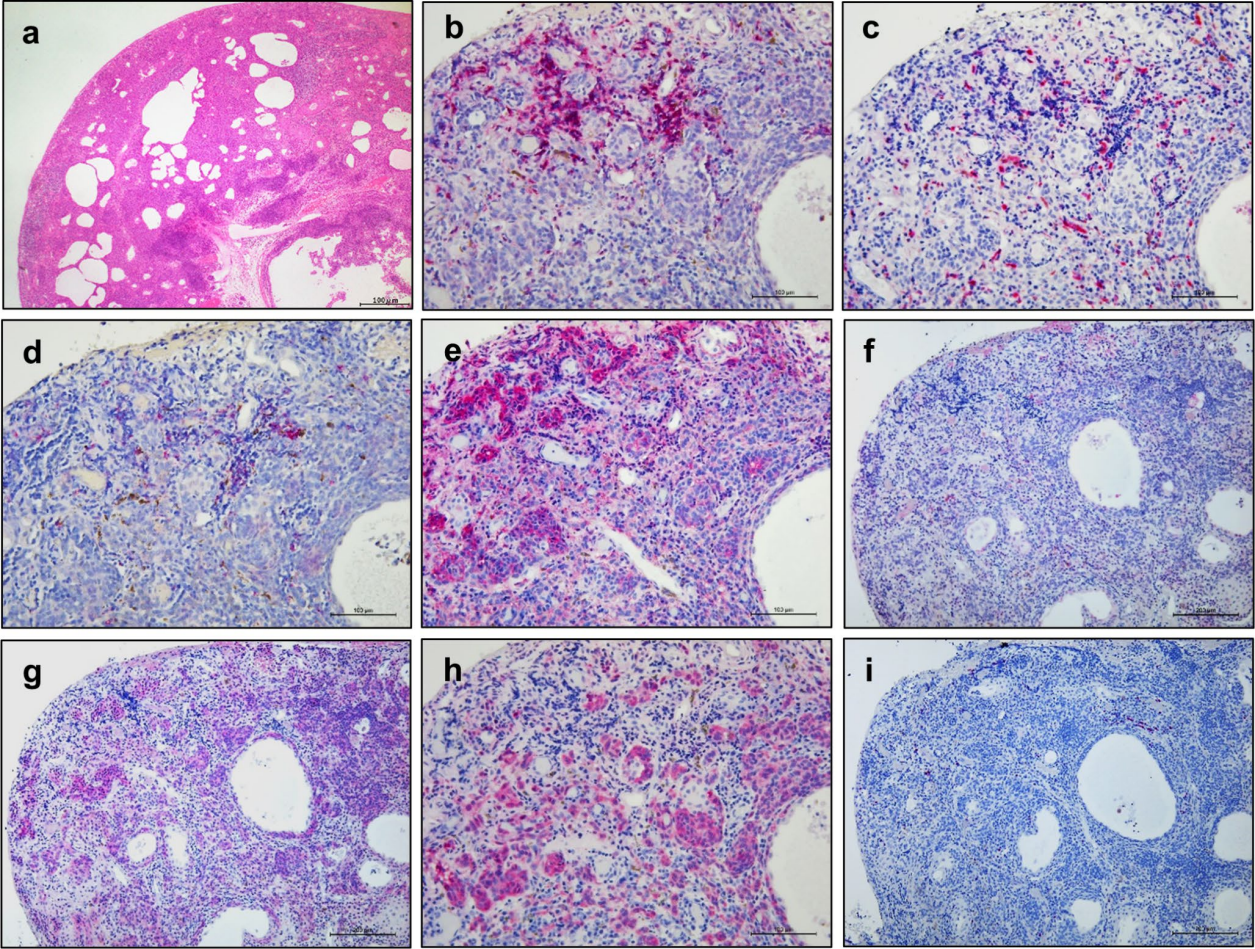
Findings of the histopathologic analyses performed for case 3. In case 3, treatment with 0.1% dexamethasone and 0.1% tacrolimus was administered for 4 months. A slight resolution of the overall lesion length and tumor diameter was observed; however, the nodular lesion with feeder vessels persisted. In the images, the red signals indicate alkaline phosphatase coloration by each antibody, whereas the brown signals indicate pigmentation. Hematoxylin and eosin staining revealed clusters of round cells with melanocytic granules in the intrinsic mucosal layer. **a**: The eosinophil infiltrate was unremarkable. **b:** CD3-positive cells and **c:** CD68-positive cells infiltrated the surrounding nevus cell clusters. **d:** A few CD20-positive cells were also observed. **e:** Staining for CD44 molecules was strongly positive. Stainings for **f:** Melan-A, **g:** melanosomes, and **h:** S100 cells were positive. **i:** In contrast, Ki-67 expression, which indicates proliferative activity, was not observed. Bar: 100 μm

Figure 4 presents the histopathologic findings of case 3: The patient was treated with 0.1% dexamethasone and 0.1% tacrolimus for 4 months. A slight resolution of the hyperemia and tumor size was observed; however, the nodular lesion with feeder vessels persisted. Therefore, the lesion was removed for cosmetic reasons. Hematoxylin and eosin staining revealed the presence of a cluster of round cells with melanocytic granules in the intrinsic mucosal layer with surrounding lymphocytes. The eosinophilic infiltration was unremarkable. The cysts were lined with multiple layers of epithelium and were found within the connective tissue (Fig. 4a). CD3-positive (T cells) and CD68-positive (macrophages) cells had infiltrated the regions surrounding the nevus cell clusters. Moreover, a few CD20-positive cells (B cells) were also observed (Fig. 4b–d). Stainings for melanoma-associated antigen (Melan A), premelanosomal glycoproteins (melanosomes), and S100 protein were positive (Fig. 4f–h). However, staining for Ki-67, a marker of proliferative activity, was negative (Fig. 4i). Staining for CD44, the adhesion molecule, was strongly positive (Fig. 4e), but that for eosinophils was not remarkable. Similar findings were observed in another resected case (case 7).

## Discussion

We clarified that IJCN is characterized by the presence of pigmented masses with trophic vessels and cystic lesions that are located near the corneal limbus of the bulbar conjunctiva. No significant differences were reported between the sexes in terms of the incidence of the nevus; however, IJCN was observed more commonly in boys with some kind of allergic reactions. Some of these characteristics are consistent with those reported in previous studies [2–5, 7–9, 13]. The serum immunoglobin E levels of boys tend to be higher than those of girls until puberty and boys tend to be more sensitive to allergies than girls [14]. This might explain the sex differences observed in the incidence of IJCN.

The triggering factors that cause marked inflammation in IJCN are not exactly known. Compared with noninflammatory nevi, IJCNs exhibit an increase in the number of eosinophils, lymphocytes, and mast cells. Fibroblasts in these lesions promote eosinophil adhesion via NGF (nerve growth factor) and induce allergic inflammation. This process is reported to be an immune response induced by the nevus itself [15]. Furthermore, Kato and colleagues reported that conjunctival melanocytes are involved in conjunctival pigmentation with a stem cell factor that is produced by fibroblasts under conditions of allergic inflammation [11]. Thus, the conjunctival pigmentation observed since early childhood in our cases might have been accelerated by recurrent inflammation of the lesions.

Conjunctival nevi rarely (< 1%) show malignant transformation [5]; however, nevus cells that are atypical or found in large nevi of > 10 mm, with acute progression, are difficult to distinguish from malignant tumors [16, 17]. Findings such as cyst formation and vascular invasion are suggestive of IJCN [2, 5, 7], and AS-OCT has been used to visualize cyst formation within the tumor and detect the border above the underlying sclera [18]. The differential diagnosis of IJCN remains challenging because vascular invasion is usually observed in both IJCN and malignant tumors [5]. We have shown that AS-OCTA can visualize low blood flow within the pathogenic lesion and the invading vessels are mainly confined to the sclera underlying the lesion. Although we consider that this observation achieved using AS-OCTA is not specific to IJCN, this is the first study to demonstrate this finding of IJCN using AS-OCTA.

That AS-OCTA can be used to differentiate IJCN from malignant tumors without any invasion, especially in children, is very important. This disease should also be differentiated from nodular scleritis. Differential diagnosis is made on the basis of clinical background factors such as age, ocular pain, sex, onset, and history of allergic conjunctivitis [19, 20]. Previously, IJCN has been characterized by the presence of epithelial inclusion cysts and solid epithelial islands [2, 6] as a variant of a benign conjunctival nevus, a subset of childhood nevi lacking maturation in particular [4, 8–10]. The present study also confirmed that IJCN is a benign melanocytic tumor that showed negative staining for Ki-67 and positive staining for Melan A and melanosomes. Infiltration of T cells and macrophages was also observed. Therefore, tacrolimus was expected to be an effective treatment for the inflammation induced by related cytokines. In our cases, the histologic findings may have been affected by the treatment with immunosuppressant eye drops; the resected tissue samples are different from those observed in the active phase. Nonetheless, we would still have observed T cells and macrophages. We hypothesized that more inflammatory cells would be observed before the administration of treatment. The inactive phase of the samples in the excised case might explain the lack of eosinophils in our cases. CD44, a cell surface glycoprotein involved in cell–cell interactions, cell adhesion, and migration, is strongly expressed in IJCN. Whether CD44 may play a role in the pathophysiology of IJCN has not been determined; however, the role of CD44 in IJCN should be clarified in the future. On the basis of the histopathologic findings of IJCN, we speculated that antiallergic agents and immunosuppressants would be effective in treating this disease. Kato and colleagues reported cases in which the use of antiallergic agents alone or in combination with tacrolimus was effective [11]. Administration of antiallergic agents is sometimes effective, but the efficacy depends on the severity and duration of the disease. All our patients were resistant to antiallergic agents and required immunosuppressive agents such as steroids and tacrolimus. However, administration of steroids may lead to complications such as glaucoma in young patients [12, 21]. In fact, two of the seven patients (28.6%) included in the present study had elevated intraocular pressure resulting from the administration of steroid eye drops. Thus, to treat IJCN, the use of immunosuppressant eye drops, such as tacrolimus or cyclosporine, is preferred over steroid eye drops. The administration of immunosuppressant eye drops was successfully tapered in most cases. However, IJCN is an allergy-related disease that occurs at a young age. Thus, treatment should be resumed if signs of recurrence are observed during follow-up.

In conclusion, the present study revealed the clinical and histopathologic characteristics of IJCN, reporting new findings obtained through AS-OCTA and the effectiveness of immunosuppressive agents in the treatment of IJCN.
